# The Minimum Dietary Diversity For Women Indicator Can Be Extended To Children And Adolescents aged 4-15 Years As A Proxy Population Indicator For Good Micronutrient Adequacy Of Diets In Low- and Middle-Income Countries

**DOI:** 10.1016/j.cdnut.2024.104508

**Published:** 2024-11-20

**Authors:** Loty Diop, Aulo Gelli, Lieven Huybregts, Joanne E Arsenault, Lilia Bliznashka, Erick Boy, Megan Deitchler, Carl Lachat, Mourad Moursi, Angelica M Ochoa-Avilés, Deanna K Olney, Elodie Becquey

**Affiliations:** 1 International Food Policy Research Institute (IFPRI), Washington, DC, United States; 2 Department of Food Technology, Safety and Health, Ghent University, Ghent, Belgium; 3 Intake–Center for Dietary Assessment at FHI360, Washington, DC, United States; 4 Department of Bioscience, Universidad de Cuenca, Loja, Cuenca, Ecuador

**Keywords:** usual intake, minimum dietary diversity, micronutrient intake adequacy, children, adolescents, low- and middle-income countries

## Abstract

**Background:**

The response to the global call for more data on children’s and adolescents’ diets and nutrition is limited by the lack of straightforward practical indicators to track their diet quality. On the basis of a food group score compiled from 10 food groups (FGS-10), the minimum dietary diversity for women, calculated as FGS-10 ≥ 5, is a validated proxy population indicator for better micronutrient intake adequacy for adult women in low- and middle-income countries (LMICs).

**Objectives:**

This study aims to validate FGS-10 and its related cutoffs against micronutrient intake adequacy in 4–15-y-old children/adolescents in LMICs.

**Methods:**

We conducted a secondary data analysis of 9 datasets of repeated 24-h recalls or weighed records including 11,524 children/adolescents aged 4–15 y, collected in 7 countries (Burkina Faso, Ghana, Ecuador, India, Malawi, Uganda, and Zambia) between 2007 and 2022. For each dataset and the pooled sample (meta-analysis), we assessed the association between FGS-10 and the mean probability of adequacy (MPA) of intake over 8 micronutrients (MPA-8), and the performance of several FGS-10 cutoffs in predicting acceptable (≥0.60) and good (≥0.80) levels of MPA-8. Robustness analyses used the 7 datasets with data on 11 micronutrients (MPA-11).

**Results:**

FGS-10 ranged from 3.0 to 4.8 across datasets, and the proportion of children/adolescents with acceptable MPA-8 ranged from 8.4% to 74%. Positive and significant associations between FGS-10 and MPA-8 were found in all datasets and the pooled sample. The optimal cutoff varied across datasets from FGS-10 ≥ 4 to FGS-10 ≥ 6. In the pooled sample, FGS-10 ≥ 5 had the highest performances in predicting acceptable and good levels of MPA-8. FGS-10 ≥ 5 was also the best proxy indicator for MPA-11 ≥ 0.80.

**Conclusions:**

The continuous FGS-10 and dichotomous FGS-10 ≥ 5 may be extended to 4–15-y-old children/adolescents in LMICs. In this population, FGS-10 ≥ 5 can be used as a proxy population indicator for good micronutrient adequacy of diets.

## Introduction

Micronutrient deficiencies increase risk of death among children and prevent them from reaching their full developmental potential [1]. They are also major contributors to the global burden of disease among children and adolescents [2]. Therefore, attention to nutrition and improvement in dietary quality are necessary in this population. This is critical given that early adolescence is a life stage during which food choices are made more independently, and dietary preferences and habits are formed, potentially influencing adult health [3].

Despite calls for more data on adolescents’ nutrition and health, most national nutrition surveys concentrate on either young children (under 5 y of age) or adult women (15 y or older). Consequently, limited evidence exists on diet quality in the 5–15-y-old population, especially in low- and middle-income countries (LMICs) [4]. Furthermore, methodological gaps exist in measuring diets in children and adolescents [5]. Large-scale dietary assessment surveys commonly use the quantitative multipass 24-h recall method validated for use in LMICs for adults self-reporting their intake or that of their young children, and to some degree in older preschoolers and adolescents [2,[6], [7], [8]]. However, the collection and utilization of quantitative 24-h recall data is challenged by high data collection costs, time constraints, data processing and analysis complexity, and limited technical capacity [9].

Hence, there is a need for accurate indicators that are easy to collect, compile, and interpret, to assess diet quality in broader age groups of children and adolescents. Simple indicators using food group scores (FGS) have been developed in response to this need, and to capture dietary diversity which is a critical contributor to diet quality. Several studies across various populations have highlighted the positive association between dietary diversity and micronutrient adequacy [[10], [11], [12]]. The minimum dietary diversity for women (MDD-W) indicator, a dichotomous indicator assessing the proportion of a population who consumed 5 of 10 food groups during the past 24 h, has been validated for adult nonpregnant and pregnant women in several settings: it was identified as a good proxy indicator for a better level of micronutrient intake adequacy in these populations [11,13]. In addition, studies assessing the validity as a proxy indicator for better micronutrient adequacy of the 5-food-group cutoff for children found that it could be extended to children in Burkina Faso (BF; 2–5 y of age), and Zambia (4–8 y) [14,15]. A recent multicountry study also concluded that the 5-food-group cutoff was a simple proxy indicator for better micronutrient adequacy (from single-day 24-h recalls) of the diet of adolescents aged 10–19 y from upper-middle- and high-income countries but suggested an adapted cutoff of 4 food groups worked better for adolescents from LMICs. However, the authors highlighted the need to validate these findings using datasets with estimates of usual intakes and across settings where adolescents have lower micronutrient adequacy levels [16].

Therefore, although MDD-W is widely used in nutritional surveys for women over 15 y, its suitability for younger children or adolescents is still questioned [17]. Our study used secondary datasets to conduct a multisite evaluation that aimed to assess the performance of a FGS based on the MDD-W food groups and related cutoffs in predicting better micronutrient adequacy (based on usual intake from repeated 24-h recalls or weighed food records) in children and adolescents aged between 4 and 15 y. In LMICs where micronutrient adequacy of the diet is an issue, and diet data are particularly scarce in school-aged children and adolescents, using simple proxies may provide essential data to decision makers and program implementers for designing and assessing programs aiming at improving diet diversity and micronutrient adequacy in these key populations.

The MDD-W food grouping was chosen because of its widespread adoption in large-scale nutrition and health surveys, its qualitative nature, and its relatively low number of food groups to focus on. This made it the simplest of a set of candidates for healthy diet metrics that include additional dimensions such as environmental sustainability of diets (EAT-Lancet validated for children over 2 y), or noncommunicable disease risk reduction [Global Diet Quality Score (GDQS), currently undergoing adaptation and validation in children aged 2–14 y] [[17], [18], [19]].

## Methods

### Study population and design

Studies conducted in LMICs, which collected food consumption data on children and adolescents aged under 16 y, using the 24-h recall or weighed records methods with several days of dietary recall for at least a subsample of respondents, were considered for inclusion. Studies retained included children and adolescents aged 4–15.9 y from 9 LMICs and were conducted between 2007 and 2022 in urban (BF1) and rural (BF2) BF, urban Ghana (Accra1 and Accra2), urban Ecuador (Ecuador), rural India (Maharashtra), rural Malawi (Malawi), rural Uganda (Uganda), and rural Zambia (Zambia) (Table 1).

**TABLE 1:** Samples description^1^.

	First recall sample size	Second recalls (%)	Reporting made by	Year(s) of data collection	Location	Male (%)	Age range (y)	Energy intake (kcal)
Accra1	755	12	Adolescent	2020–2021	Urban	46	12–15	2391 ± 1065
Accra2	71	100	Adolescent	2021–2022	Urban	37	9.0–15	2445 ± 1098
BF1	237	26	Adolescent	2019	Urban	47	10–14	1775 ± 700
BF2	2586	18	Caregiver	2017, 2019	Rural	51	4.0–8.5	1453 ± 644
Ecuador	3550	48	Adolescent	2010, 2011, 2012	Urban	37	10–15	1838 ± 668
Maharashtra	219	50	Caregiver	2010	Rural	60	4.0–5.0	1001 ± 399
Malawi	3605	6.3	Caregiver	2015, 2016	Rural	51	4.0–8.0	1474 ± 915
Uganda	367	16	Caregiver	2007, 2008	Rural	51	4.0–6.4	1695 ± 583
Zambia	134	100	Caregiver	2009	Urban and rural	43	4.0–5.6	1424 ± 557
Pooled	11524	25	—	—	—	46	4.0–15	1651 ± 825

All data collected by 24-h recall, except for Accra2 collected by weighed record. Values of energy intakes are related to day 1 of the actual intakes.

Abbreviation: BF, Burkina Faso.

1 Values are mean ± SD, percentages (%) or frequencies (*n*).

Detailed information on the individual studies’ primary objectives, design, setting, and ethical clearance has been published elsewhere [7,[20], [21], [22], [23], [24], [25], [26], [27]]. Briefly, these studies were either observational studies aiming at validating data collection methods or describing dietary patterns; or impact evaluations of interventions to improve children’s diets, cognitive development, adolescents’ physical fitness, or micronutrient intake through behavioral interventions, biofortification or promotion of nutritious crop cultivation. Despite being collected in the same urban area, Ghana’s capital city Accra, the Accra1 and Accra2 datasets were collected from different populations. The Accra1 dataset consisted of adolescents from poor and middle-class neighborhoods, whereas the Accra2 study enrolled adolescents from all socioeconomic classes in Greater Accra region (including higher socioeconomic areas) [21,22].

### Dietary data collection

Eight of the 9 datasets included in this study used a quantitative multipass 24-h recall [6]. One study (Accra2) used observed weighed records covering the first meal to the last meal of the day. When recipes of foods were unknown, standard recipes were collected and used to provide food composition [28]. As several of the individual studies involved children under 7 y (BF2, Maharashtra, Uganda, and Zambia), their caregivers provided information on dietary intake. In studies with older children between 7 and 10 y (BF2 and Malawi), the study enumerator requested the child to be present during their caregiver report whenever possible. This allowed the child to report what and how much they ate at school or at other moments when their caregiver was absent. In studies involving adolescents aged 11–15 y (Accra1, Accra2, BF1, and Ecuador), reporting was made by the adolescents themselves.

All datasets had second recalls on a nonconsecutive day, with the proportion of second recall availability ranging from 6% to 100% (Table 1).

For all datasets except Malawi, the conversion of raw food intakes into micronutrient intakes using appropriate food composition tables was already completed [[24], [25], [26], [27],^29^,^30^]. Micronutrient intakes were calculated using the Malawian 2019 food composition table for the Malawi dataset [31]. Possible over- or under-reporters not resulting from obvious reporting errors were not excluded from any dataset [32].

### Dietary diversity score

Every food item reported during the first 24-h recall or weighed record was classified into 1 of the following MDD-W food groups: *1*) starchy staples (grains, white roots and tubers, and plantains), *2*) pulses, *3*) nuts and seeds, *4*) dairy, *5*) flesh foods, *6*) eggs, *7*) dark-green leafy vegetables, *8*) vitamin A-rich fruits and vegetables, *9*) other vegetables, *10*) other fruits, and *11*) other foods [17]. Food items weighing <15 g were considered condiments or seasoning and not classified in any group, as recommended by MDD-W food grouping guidelines [17]. For mixed dishes, recipes provided by the respondent or standard recipes were used to estimate the proportions of each food group consumed. Subsequently, the FGS-10 was calculated for the first recall day for each child/adolescent. FGS-10 summed the number of food groups consumed among the first 10 food groups listed above. Dichotomous indicators related to various cutoffs were calculated and coded as 1 if FGS-10 was greater than or equal to a given cutoff, and 0 otherwise [17].

### Micronutrient adequacy

Seven (Accra1, Accra2, BF1, BF2, Malawi, Uganda, and Zambia) of the 9 datasets contained information on the following 11 micronutrients: iron, calcium, zinc, thiamine, niacin, riboflavin, folate and vitamins A, C, B-6, and B-12. Ecuador and Maharashtra did not contain measures of vitamins B-6 and B-12 (Ecuador and Maharashtra) or folate (Ecuador only), because of the unavailability of this information in the food composition tables used to compile micronutrient intakes. Therefore, micronutrient adequacy was calculated considering *1*) 8 micronutrients available across all 9 datasets; and *2*) 11 micronutrients available in 7 datasets.

Micronutrient intakes were expressed in μg or mg/d, except for vitamin A expressed in retinol activity equivalents. Probabilities of adequacy (PA) of micronutrient intake were calculated using the probability approach [33]. Estimated average requirements and SD for children and adolescents were based on harmonized recommendations, considering semiunrefined diets for zinc bioavailability [34].

For all micronutrients, we applied an adapted version of the methodology used in the validation of MDD-W in nonpregnant and pregnant women in LMICs [10,11,13]. Briefly, we employed a Box–Cox transformation to obtain symmetrical distributions of nutrient intakes and, then, calculated the best linear unbiased predictor of a child/adolescent’s usual intake, using the intraindividual and interindividual variances [35]. The PA of the intake of each micronutrient, except iron, was then estimated as the probability that a child/adolescent’s usual intake was above the requirement for that micronutrient along the normal distribution of requirements [33]. Considering the skewness of the distribution of iron requirements, not clearly considered in the harmonized average requirement (H-AR) for iron, a modified version of the Institute of Medicine (IOM) tables was used to retrieve PA. The modification consisted of applying to the IOM tables a correction factor that was the difference between the IOM’s estimated average requirement and the H-AR, and adjusting the tables to various levels of bioavailability. Considering the different levels of consumption of flesh foods in the study sites, we applied a level of 5% of bioavailability for sites with a low proportion of consumption of flesh foods (BF2 and Maharashtra), 10% for sites with a moderate proportion of consumption (BF1, Malawi, Uganda, and Zambia), and 16% for sites with high proportion of consumption (Accra1, Accra2, and Ecuador) [36].

Overall micronutrient adequacy was assessed through the mean probability of adequacy (MPA), calculated for each child/adolescent by averaging the PAs values across 8 micronutrients (MPA-8). All analyses were then replicated using an MPA calculated using 11 micronutrients (MPA-11) for the 7 datasets for which it was possible, to assess the robustness of the findings.

### Statistical analyses

Pooled and site-specific analyses were conducted. The latter allowed us to describe the specifics of each site in terms of diet and to assess the validity of FGS-10 and various cutoffs for each of these sites, whereas the pooled analysis aimed at giving a summary of their predictive performances.

We first assessed the association between FGS-10 and MPA-8 both visually and using correlation coefficients. The visual method consisted of plotting the average MPA-8 of children/adolescents with different levels of FGS-10 in each site and the pooled sample. Because the distributions of MPA-8 were skewed in most sites, the correlation between FGS-10 and MPA-8 was assessed using Spearman rank correlation coefficients, with and without controlling for energy intake [37]. The significance level was set at 0.05. All correlation analyses were replicated for MPA-11. Additionally, visual and correlation analyses were done separately for different age ranges (4–4.9 y, 5–9.9 y, and 10–15.9 y) to confirm the consistency of the association across age groups.

Second, we evaluated the performance of FGS-10 as a predictor of better levels of micronutrient adequacy in children and adolescents. Considering the small proportion of children/adolescents reaching the ideal level of MPA = 1 (0.31% of the pooled sample), lower MPA cutoffs were considered. We defined MPA-8 ≥ 0.60 as an acceptable level of MPA. This threshold was considered because it was used in previous validation studies of MDD-W, justified by distributions of MPA in the samples which did not allow authors to use higher levels of MPA [11,13]. In our sample, as the proportion of children/adolescents reaching an MPA-8 ≥ 0.60 was higher than 15% in most sites and was even above 60% in 3 sites, we additionally defined MPA-8 ≥ 0.80 as a good level of MPA (Table 2). The performances of FGS-10 and its related cutoffs were assessed both as predictors of acceptable (MPA-8 ≥ 0.60) and good (MPA-8 ≥ 0.80) levels of micronutrient adequacy to assess the validity of our conclusions when considering the level used to validate MDD-W for women, but also consider the highest level of MPA allowed by our samples. The prediction performances of FGS-10 were assessed by calculating the AUC for MPA-8 ≥ 0.60 and MPA-8 ≥ 0.80. AUC values below 0.70 were considered as depicting poor classification performances. AUC values equal or over 0.70, 0.80, and 0.90 were considered acceptable, excellent, and outstanding classification performances, respectively [38].

**TABLE 2:** Food group scores and prevalence of adequate intake of 11 micronutrients and mean probability of adequacy (MPA)^1^.

	Accra1, 12–15 y (*n* = 755)	Accra2, 9–15 y (*n* = 71)	BF1, 10–14 y (*n* = 237)	BF2, 4–8.5 y (*n* = 2586)	Ecuador, 10–15 y (*n* = 3550)	Maharashtra, 4–5 y (*n* = 219)	Malawi, 4–8 y (*n* = 3605)	Uganda, 4–6 y (*n* = 367)	Zambia, 4–5 y (*n* = 134)	Pooled, 4–15 y (*n* = 11524)
FGS-10	3.8 ± 1.3	4.3 ± 1.3	3.9 ± 1.1	3.0 ± 1.0	4.8 ± 1.3	3.7 ± 1.4	3.5 ± 1.2	4.5 ± 1.4	4.6 ± 1.2	3.9 ± 1.4
FGS-10 ≥4 (%)	59	70	59	28	83	46	48	75	79	57
FGS-10 ≥5 (%)	29	48	28	7.6	58	28	20	45	54	31
FGS-10 ≥6 (%)	11	17	7.2	0.93	30	18	6.2	22	19	14
PA of iron	0.96 ± 0.14	0.98 ± 0.7.9	0.76 ± 0.29	0.62 ± 0.25	0.93 ± 0.15	0.32 ± 0.15	0.58 ± 0.22	0.82 ± 0.14	0.80 ± 0.15	0.74 ± 0.27
PA of zinc	0.40 ± 0.42	0.37 ± 0.43	0.16 ± 0.30	0.72 ± 0.39	0.11 ± 0.25	0.33 ± 0.38	0.25 ± 0.39	0.72 ± 0.40	0.69 ± 0.38	0.34 ± 0.43
PA of calcium	0.0040 ± 0.061	0.21 ± 0.36	0.0032 ± 0.036	0.036 ± 0.17	0.051 ± 0.18	0.18 ± 0.35	0.086 ± 0.26	0.029 ± 0.16	0.0075 ± 0.086	0.057 ± 0.21
PA of vitamin A	0.22 ± 0.39	0.85 ± 0.32	0.19 ± 0.33	0.17 ± 0.36	0.51 ± 0.44	0.12 ± 0.29	0.19 ± 0.37	0.55 ± 0.48	0.77 ± 0.40	0.31 ± 0.43
PA of vitamin C	0.28 ± 0.42	0.84 ± 0.34	0.60 ± 0.46	0.30 ± 0.45	0.35 ± 0.45	0.24 ± 0.41	0.56 ± 0.48	0.98 ± 0.13	0.87 ± 0.31	0.43 ± 0.47
PA of thiamin	0.45 ± 0.43	0.86 ± 0.30	0.28 ± 0.34	0.51 ± 0.45	0.77 ± 0.38	0.43 ± 0.39	0.68 ± 0.42	0.94 ± 0.21	0.85 ± 0.32	0.65 ± 0.43
PA of riboflavin	0.10 ± 0.23	0.081 ± 0.19	0.10 ± 0.24	0.19 ± 0.36	0.29 ± 0.38	0.53 ± 0.47	0.060 ± 0.22	0.54 ± 0.45	0.55 ± 0.45	0.19 ± 0.35
PA of niacin	0.71 ± 0.38	0.91 ± 0.23	0.48 ± 0.37	0.32 ± 0.39	0.88 ± 0.26	0.57 ± 0.40	0.23 ± 0.36	0.88 ± 0.27	0.84 ± 0.30	0.53 ± 0.44
PA of folate	0.39 ± 0.41	0.81 ± 0.34	0.41 ± 0.41	0.51 ± 0.46	—	—	0.59 ± 0.45	0.95 ± 0.17	0.88 ± 0.27	0.55 ± 0.45
PA of vitamin B_6_	0.49 ± 0.45	0.90 ± 0.27	0.32 ± 0.42	0.47 ± 0.45	—	—	0.76 ± 0.39	0.99 ± 0.7.5	0.95 ± 0.19	0.65 ± 0.44
PA of vitamin B_12_	0.79 ± 0.37	0.56 ± 0.45	0.20 ± 0.36	0.056 ± 0.23	—	—	0.14 ± 0.34	0.32 ± 0.45	0.28 ± 0.44	0.19 ± 0.38
MPA-8	0.39 ± 0.20	0.64 ± 0.18	0.32 ± 0.19	0.36 ± 0.24	0.49 ± 0.19	0.34 ± 0.27	0.33 ± 0.22	0.68 ± 0.18	0.67 ± 0.19	0.41 ± 0.23
MPA-8 ≥0.60 (%)	18	66	8.4	18	29	16	16	74	69	22
MPA-8 ≥0.80 (%)	2.1	18	1.7	5.9	5.6	8.9	3.6	31	35	6.0
MPA-11	0.44 ± 0.22	0.67 ± 0.18	0.32 ± 0.20	0.36 ± 0.24	—	—	0.38 ± 0.22	0.70 ± 0.15	0.68 ± 0.17	0.40 ± 0.24
MPA-11 ≥0.60 (%)	26	68	9.7	17	—	—	12	81	75	22
MPA-11 ≥0.80 (%)	4.2	23	1.7	4.3	—	—	4.1	30	26	5.9

Cells with (–) indicate that the compilation of the prevalence of adequacy or MPA was not possible for the setting considered.

Abbreviations: BF, Burkina Faso; FGS-10, food group score based on the minimum dietary diversity for women (MDD-W) indicator guidelines; MPA-8, mean probability of adequacy calculated on 8 micronutrients; MPA-11, mean probability of adequacy calculated on 11 micronutrients; PA, probability of adequacy.

1 Values are mean ± SD, proportions or percentages.

Finally, sensitivity, specificity, and percentage of correct classification (PCC) were calculated for various FGS-10 cutoffs (including FGS-10 ≥ 5) to assess their performances as predictors of acceptable or good levels of MPA. Sensitivity was defined as the percentage of children/adolescents with FGS-10 higher than a given cutoff (5 groups, for example) within children/adolescents with an acceptable (MPA-8 ≥ 0.60) or good (MPA-8 ≥ 0.80) level of MPA. Specificity was defined as the percentage of children/adolescents whose FGS-10 was lower than a given cutoff (5 groups, for example) within children/adolescents with an unacceptable (MPA-8 < 0.60) or bad (MPA-8 < 0.80) level of MPA. The PCC was defined as the percentage of children/adolescents correctly classified as having acceptable, good, unacceptable, or bad MPA by a given FGS-10 cutoff.

Building on previous validation studies, an FGS-10 cutoff was considered to have fair predictive performance when PCC > 0.60 and both specificity and sensitivity >0.50, with ≥1 of them being >0.60 [13]. An FGS-10 cutoff was considered to have good predictive performance when PCC >0.70, and both sensitivity and specificity >0.60. For each site and the pooled sample, we assessed the feasibility of identifying fair or good FGS-10 cutoffs and the optimal cutoff was identified as the cutoff with the best predictive performance. When several FGS-10 cutoffs had similar fair or good predictive performances, the optimal cutoff was defined as the cutoff with the highest Youden index, calculated as Sensitivity + Specificity – 1. However, when no fair or good FGS-10 cutoff could be identified for a given site, we concluded that no acceptable cutoff could be identified for that site. The same methodology was applied using MPA-11 to verify the robustness of results when considering 3 additional micronutrients (folate, vitamins B-6, and B-12) in the 7 datasets for which they were available so that it could be directly comparable with previous MDD-W validation results.

Because sample sizes varied widely across datasets (Table 1), we performed a pooled analysis in the form of a meta-analysis of the correlation coefficients, sensitivities, and specificities resulting from the site-specific analyses, using inverse variance weighing and Hartung and Knapp adjustments to estimate the pooled correlation coefficients [[39], [40], [41]]; and summary receiver operating characteristics analysis and Reitsma bivariate regression models to estimate pooled AUC, sensitivities, and specificities [42,43]. We applied the same definitions of fair and good FGS-10 cutoff as the ones used for the site-specific analyses. The only difference was that pooled AUC value was used instead of the PCC, as pooled PCC was not an output of the regression models used. This pooled analysis was conducted using MPA-8 and repeated using MPA-11 to assess the robustness of the findings.

## Results

### Sample description

Adolescents were mostly located in urban areas (Accra1, Accra2, BF1, and Ecuador), whereas children were mainly from rural areas (BF2, Maharashtra, Malawi, and Uganda) (Table 1). In children aged under 5 y, energy intake of the first recall day was lowest in Maharashtra (1001 kcal) and highest in Uganda (1695 kcal). Considering older children/adolescents, BF1 (1775 kcal) had the lowest energy intake whereas the highest intakes were found in Accra1 (2391 kcal) and Accra2 (2445 kcal).

### Consumption of MDD-W-related food groups

In all sites, the most frequently (96%–100%) consumed MDD-W food group on the first recall day was starchy staples (Supplemental Table 1). The consumption of the remaining food groups varied significantly between sites, with some being frequently consumed in certain locations and rarely in others. The average dietary diversity score in the pooled sample was 3.9 and ranged between 3.0 and 4.8 across sites (Table 2). In datasets with younger children (<10 y), the highest average dietary diversity scores were observed in Zambia (FGS-10 = 4.6) and Uganda (4.5), whereas BF2 had the lowest levels (3.0). Among adolescents, the highest level was found in Ecuador (4.8), whereas the lowest were in Accra1 (3.8) and BF1 (3.9).

### Adequacy of micronutrient intake

Considering the MPA calculated over the 8 micronutrients available in all the datasets (MPA-8), the highest average levels of MPA-8 in younger children were found in Uganda (0.68), whereas the lowest level was found in Malawi (0.33) (Table 2). Among adolescents, the highest levels of MPA-8 were found in Accra2 (0.64) and Ecuador (0.49). The proportion of children/adolescents with an acceptable level of micronutrient adequacy (MPA-8 ≥ 0.60) ranged from 8.4% to 74%, with the lowest levels found in BF1, and the highest in Uganda. Values of MPA-11 were close to those of MPA-8 for each site, with differences no higher than 5 percentage points (Table 2).

### Association between dietary diversity and micronutrient adequacy

**FIGURE 1:**
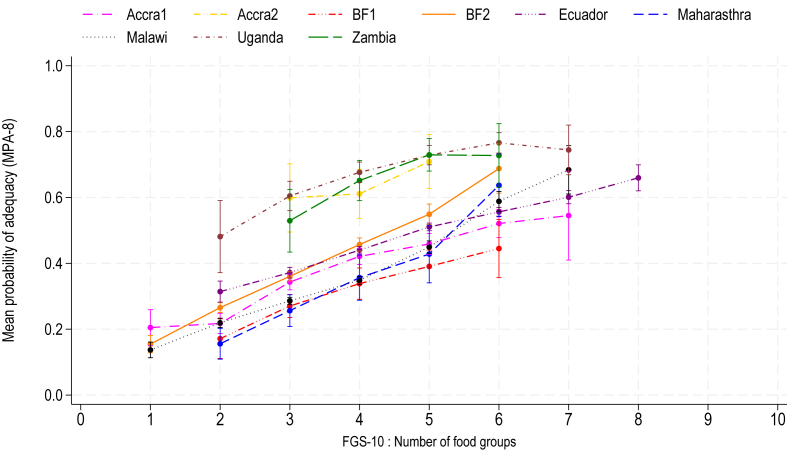
Mean probability of adequacy calculated on 8 micronutrients (MPA-8) by FGS-10, for children and adolescents aged 4–15 y. Values are mean ± SEM. Food group scores with <10 observations were excluded. Accra1 = 755, Accra2 = 71, BF1 = 237, BF2 = 2586, Ecuador = 3550, Maharashtra = 219, Malawi = 3605, Uganda = 367, Zambia = 134. BF, Burkina Faso; FGS-10, food group score compiled from 10 food groups.

**FIGURE 2:**
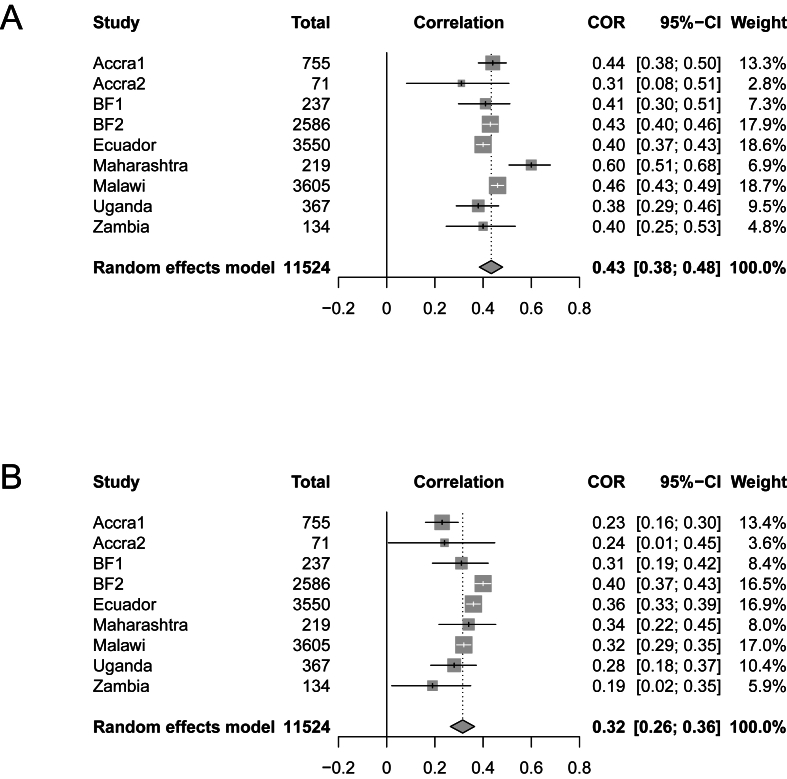
Correlation between FGS-10 and MPA-8 not adjusting for energy intake (A) and adjusting for energy intake (B). Values are Spearman’s rank correlation coefficients, pooled correlation coefficients and confidence intervals (CIs) of the pooled coefficients. Values adjusted for energy intake are partial Spearman’s rank correlation coefficients. BF, Burkina Faso; COR, correlation; FGS-10, food group score compiled from 10 food groups; MPA-8, mean probability of adequacy calculated on 8 micronutrients.

The association was first assessed visually, and positive associations were identified between FGS-10 and MPA-8 in all sites (Figure 1). This was confirmed by the positive significant correlation coefficients between MPA-8 and FGS-10, ranging from 0.31 to 0.60, found for each site and in the pooled sample (Figure 2). As expected, adjusting for energy intake generally attenuated the associations, but all coefficients remained statistically significant. Results were consistent for MPA-11 (Supplemental Figure 1). Results by child age group were consistent with positive associations between dietary diversity and micronutrient adequacy in all 3 age groups (Supplemental Figures 2 and 3).

### Identification of acceptable and optimal cutoffs

**FIGURE 3:**
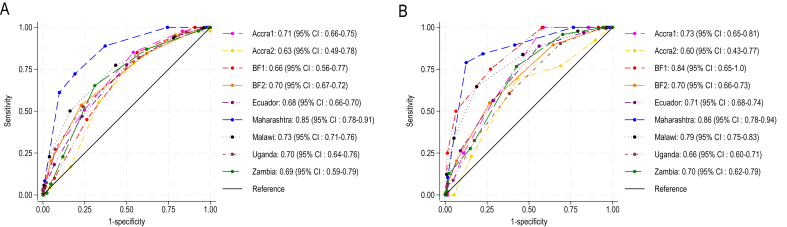
Receiver operating characteristic curves of FGS-10 predicting MPA-8 ≥0.60 (A) and MPA-8 ≥0.80 (B) by study site. BF, Burkina Faso; CI, confidence interval; FGS-10, food group score compiled from 10 food groups; MPA-8, mean probability of adequacy calculated on 8 micronutrients.

Site-specific AUC ranged from 0.63 [95% confidence interval (CI): 0.49, 0.78] in Accra2 to 0.85 (95% CI: 0.78, 0.91) in India (Figure 3). The performances of FGS-10 as a predictor of MPA-8 ≥ 0.60 were excellent (AUC > 0.80) in Maharashtra, acceptable (AUC: 0.70–0.80) in Accra1, BF2, Malawi, and Uganda, and poor (AUC < 0.70) in Accra2, BF1, Ecuador, and Zambia. Performances were generally improved in individual sites for the prediction of MPA-8 ≥ 0.80. For most sites, AUC values were similar or higher using MPA-11, showing that FGS-10 had even better performances when predicting a more comprehensive indicator of micronutrient adequacy (Supplemental Figure 4).

In the pooled sample, the FGS-10 ≥ 5 was the best FGS-10 cutoff in predicting MPA-8 ≥ 0.60 with a fair predictive performance: pooled sensitivity of 55% (95% CI: 43%, 66%), pooled specificity of 77% (95% CI: 67%, 86%), and pooled AUC of 0.71 (Table 3). In site-specific analyses, FGS-10 ≥ 5 was the best cutoff in Accra1, Maharashtra, Malawi, and Zambia. FGS-10 ≥ 4 had fair predictive performances and was the best cutoff for BF2. No fair or good cutoff was identified in the remaining sites. Considering the prediction of a higher level of micronutrient adequacy, MPA-8 ≥ 0.80, FGS-10 ≥ 5 had good-to-fair performances and was the best proxy for higher micronutrient adequacy in the pooled sample, Accra1, Malawi, Uganda, and Zambia (Table 4). A higher cutoff (FGS-10 ≥ 6) was the best in Ecuador and Maharashtra, whereas FGS-10 ≥ 4 worked better for BF1 and BF2.

**TABLE 3:** Summary of FGS-10 cutoffs characteristics relative to predicting MPA-8 ≥0.60^1^.

	Sensitivity (%)	Specificity (%)	Youden index	PCC (%)/pooled AUC
Accra1				
FGS-10 ≥4	85	46	0.31	53
FGS-10 ≥5 (MDD-W)^2^^,^^4^	51	75	0.26	71
FGS-10 ≥6	27	92	0.19	81
Accra2				
FGS-10 ≥4	79	46	0.25	68
FGS-10 ≥5 (MDD-W)	55	67	0.22	59
FGS-10 ≥6	17	83	0.00	39
BF1				
FGS-10 ≥4	85	44	0.29	47
FGS-10 ≥5 (MDD-W)	45	74	0.19	71
FGS-10 ≥6	10	93	0.03	86
BF2				
FGS-10 ≥4^2^^,^^4^	54	77	0.31	73
FGS-10 ≥5 (MDD-W)	20	95	0.16	82
FGS-10 ≥6	4.0	100	0.04	83
Ecuador				
FGS-10 ≥4	95	21	0.16	42
FGS-10 ≥5 (MDD-W)	78	50	0.28	58
FGS-10 ≥6	47	77	0.24	68
Maharashtra				
FGS-10 ≥4^2^	89	63	0.52	67
FGS-10 ≥5 (MDD-W)^3^^,^^4^	72	81	0.53	80
FGS-10 ≥6^3^	61	90	0.51	85
Malawi				
FGS-10 ≥4	77	57	0.34	59
FGS-10 ≥5 (MDD-W)^2^^,^^4^	50	84	0.34	80
FGS-10 ≥6	23	96	0.19	87
Uganda				
FGS-10 ≥4	82	43	0.25	72
FGS-10 ≥5 (MDD-W)	53	76	0.29	59
FGS-10 ≥6	27	93	0.20	44
Zambia				
FGS-10 ≥4	87	38	0.25	72
FGS-10 ≥5 (MDD-W)^2^^,^^4^	65	69	0.34	66
FGS-10 ≥6	23	88	0.11	43
Pooled				
FGS-10 ≥4	82, CI: 73, 88	49, CI: 38, 60	0.31, CI: 0.26, 0.35	0.72
FGS-10 ≥5 (MDD-W)^2^^,^^4^	55, CI: 43, 66	77, CI: 67, 86	0.32, CI: 0.28,0.35	0.71
FGS-10 ≥6	25, CI: 15, 38	9,3 CI: 86, 97	0.18, CI: 0.11, 0.26	0.65

Abbreviations: BF, Burkina Faso; CI, confidence interval; FGS-10, food group score based on the minimum dietary diversity for women (MDD-W) indicator guidelines; MPA-8, mean probability of adequacy calculated over 8 micronutrients; PCC, percentage of correct classification; Pooled AUC, Area under the curve obtained by plotting sensitivities and false positive rates of all sites.

1 Values of sensitivity, specificity, and correctly classified are percentages.

2 Cutoff with fair predictive performances, defined as PCC >60% and both specificity and sensitivity >50%, with ≥1 of them being >60%.

3 Cutoff with good predictive performances, defined as PCC >70%, and both sensitivity and specificity >60%.

4 Optimal cutoff, defined as cutoff with the highest Youden index (sensitivity + specificity – 1).

**TABLE 4:** Summary of FGS-10 cutoffs characteristics relative to predicting MPA-8 ≥0.80^1^.

	Sensitivity (%)	Specificity (%)	Youden index	PCC (%)/pooled AUC
Accra1				
FGS-10 ≥4	100	41	0.41	43
FGS-10≥5 (MDD-W)^2^^,^^4^	56	71	0.27	71
FGS-10 ≥6	25	89	0.14	88
Accra2				
FGS-10 ≥4	77	31	0.08	39
FGS-10 ≥5 (MDD-W)	69	57	0.26	59
FGS-10 ≥6	23	85	0.08	73
BF1				
FGS-10≥4^3^^,^^4^	75	73	0.48	73
FGS-10≥5 (MDD-W)^2^	50	94	0.44	93
FGS-10 ≥6	25	99	0.24	98
BF2				
FGS-10 ≥4^2^^,^^4^	55	74	0.28	72
FGS-10 ≥5 (MDD-W)	20	93	0.14	89
FGS-10 ≥6	4.6	99	0.04	94
Ecuador				
FGS-10 ≥4	97	17	0.14	22
FGS-10 ≥5 (MDD-W)	89	44	0.33	46
FGS-10 ≥6^2^^,^^4^	56	72	0.28	71
Maharashtra				
FGS-10 ≥4^2^	90	59	0.48	61
FGS-10 ≥5 (MDD-W)^3^	84	78	0.62	78
FGS-10 ≥6^3^^,^^4^	79	88	0.66	87
Malawi				
FGS-10 ≥4	84	54	0.38	55
FGS-10 ≥5 (MDD-W)^3^^,^^4^	65	81	0.46	81
FGS-10 ≥6	34	95	0.29	93
Uganda				
FGS-10≥4	90	31	0.22	50
FGS-10 ≥5 (MDD-W)^2^^,^^4^	61	62	0.22	61
FGS-10 ≥6	33	83	0.15	67
Zambia				
FGS-10 ≥4	96	30	0.26	53
FGS-10 ≥5 (MDD-W)^2^^,^^4^	77	58	0.34	64
FGS-10 ≥6	28	85	0.13	65
Pooled				
FGS-10 ≥4	87, CI: 78, 93	42, CI: 31, 55	0.29, CI: 0.23, 0.35	0.71
FGS-10 ≥5 (MDD-W)^3^^,^^4^	66, CI: 50, 79	71, CI: 59, 81	0.36, CI: 0.27, 0.44	0.74
FGS-10 ≥6	32, CI: 19, 49	90, CI: 82, 96	0.23, CI: 0.12, 0.35	0.71

Abbreviations: BF, Burkina Faso; CI, confidence interval; FGS-10, food group score based on the minimum dietary diversity for women (MDD-W) indicator guidelines; MPA-8, mean probability of adequacy calculated over 8 micronutrients; PCC, percentage of correct classification; Pooled AUC, Area under the curve obtained by plotting sensitivities and false positive rates of all sites.

1 Values of sensitivity, specificity, and correctly classified are percentages.

2 Cutoff with fair predictive performances, defined as PCC >60% and both specificity and sensitivity >50%, with ≥1 of them being >60%.

3 Cutoff with good predictive performances, defined as PCC >70%, and both sensitivity and specificity >60%.

4 Optimal cutoff, defined as cutoff with the highest Youden index (sensitivity + specificity – 1).

In sensitivity analyses using MPA-11, FGS-10 ≥ 4 was the best predictor of MPA-11 ≥ 0.60 in the pooled sample, BF2, and Uganda (Supplemental Table 2). However, FGS-10 ≥ 5 performed more accurately in predicting MPA-11 ≥ 0.80 in the pooled sample, Accra1, Malawi, and Uganda (Supplemental Table 3).

## Discussion

This study examined whether an FGS based on the MDD-W classification (FGS-10) and its related cutoffs can be used as proxies for micronutrient intake adequacy in children and adolescents aged 4–15 y. We found that the continuous FGS-10 was positively associated with micronutrient intake adequacy, and that FGS-10 ≥ 5 was a fair proxy indicator for an acceptable level of micronutrient intake adequacy (MPA-8 ≥ 0.60), and a fair-to-good proxy indicator of good micronutrient intake adequacy (MPA-8 ≥ 0.80) in children and adolescents aged 4–15 y overall, although some heterogeneity was observed. These findings are consistent with emerging literature suggesting that the use of diet diversity metrics can be extended from women of reproductive age to children and adolescents in LMICs, because they are fair proxy indicators for better micronutrient adequacy of the diet at population level [[14], [15], [16]].

Similar to research in other populations [11,13,16], moderate predictive performances show that MDD-W is not a good individual-level screening or diagnosis tool to predict acceptable or good micronutrient intake adequacy at the individual level. However, the consistent positive correlations between FGS-10 and micronutrient intake adequacy, and the acceptable discriminatory power of FGS-10 (reflected in AUC) to identify fair or good micronutrient intake adequacy in most sites, indicate that FGS-10 may be extended to children and adolescents aged 4–15 y, for population-level assessment of dietary diversity as a predictor of micronutrient intake adequacy. FGS-10 may also be used for performance assessments of programs with a focus on improving children’s diets, in situations where using more precise but resource-intensive tools is not justified after careful balance of objective, time, capacity, and budget. Additionally, reporting on the distribution of the continuous FGS-10 allows us to capture the variation in the number of food groups consumed within a population, whereas the detailed tabulation of food group data can be useful for policymakers to identify underconsumed healthy food groups. Further research is still needed to confirm the potential of FGS-10 for monitoring micronutrient adequacy over time. Although emerging evidence confirms the robustness of predictive performances across seasons [14,15], in the absence of longitudinal data, we could not characterize the performance of FGS-10 to reflect changes in micronutrient adequacy across seasons and year.

Because nutrition policies or programs often value the use of a dichotomous indicator to evaluate risk of inadequate intake at population level (because this can be translated into a burden), we assessed the performance of several FGS-10 cutoffs as proxy indicators for acceptable or good micronutrient intake adequacy. In the pooled sample, FGS-10 ≥ 5 emerged as the best proxy indicator for good or acceptable adequacy of intake (MPA-11 ≥ 0.80, MPA-8 ≥ 0.80, and MPA-8 ≥ 0.60), although FGS-10 ≥ 4 was the optimal proxy of acceptable adequacy of micronutrient intake across 11 micronutrients (MPA-11 ≥ 0.60).

**FIGURE 4:**
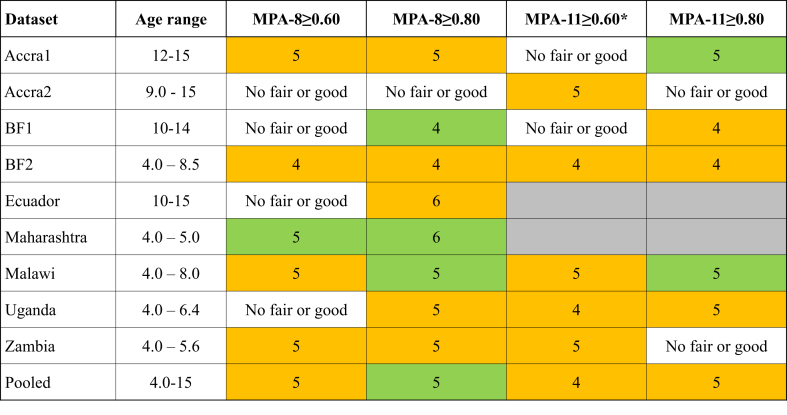
Summary of optimal FGS-10 cutoffs identified for each individual study site and in the pooled sample. Orange: fair cutoff: PCC >60%; sensitivity and specificity both >50% and ≥1 of them >60%. Green: good cutoff: PCC >70%; sensitivity >60%; specificity >60%. Gray: MPA-11 not compiled for this dataset. When several cutoffs had the same predictive performances, the one with the highest Youden index (sensitivity + specificity – 1) was selected. ∗MPA definition and level comparable with previous validation studies on adult women and adolescents [11,13,16]. BF, Burkina Faso; FGS-10, food group score compiled from 10 food groups; MPA, mean probability of adequacy; MPA-8, mean probability of adequacy calculated on 8 micronutrients; MPA-11, mean probability of adequacy calculated on 11 micronutrients; PCC, percentage of correct classification.

Some important heterogeneity across sites in our study triggers the question of whether cutoffs should be adapted for some contexts (Figure 4). Ecuador required a higher cutoff (FGS-10 ≥ 6) for the prediction of a good level of MPA (MPA-8 ≥ 0.80), whereas in urban (BF1) and rural (BF2) BF, FGS-10 ≥ 4 was found to consistently be the best cutoff. The last finding was consistent with validation findings on populations of young children and women of reproductive age in the same study, which showed that FGS-10 ≥ 4 was optimal for this context [15]. Other multicountry validations have found similar heterogeneities across populations [11,13,16]. To our knowledge, 1 [44] of 3 studies [15,45] aiming to identify an optimal MDD-W-related cutoff found a different cutoff (FGS-10 ≥ 6 for adolescents in the United States). It is beyond the scope of the present analysis to identify the characteristics that explain this heterogeneity; however, if a dichotomous indicator is deemed necessary by practitioners, we conclude that FGS-10 ≥ 5 seems to be a good compromise between FGS-10 ≥ 4 and FGS-10 ≥ 6 for global use as population proxy indicator for better micronutrient adequacy in children and adolescents aged 4–15 y in LMICs. However, in this age group, although compiled the same way as the standard MDD-W is, FGS-10 ≥ 5 performed better at reflecting a higher level of micronutrient adequacy (MPA-11 ≥ 0.8) than the level of micronutrient adequacy predicted fairly well in women (MPA-11 ≥ 0.6) [11,13]. In specific settings where a different cutoff has been consistently found, as this is the case for BF, stakeholders might consider using a site-specific cutoff that might provide more accurate results.

Our findings are comparable with those of a recent multicountry study in adolescents aged 10–19 y in 18 countries that identified FGS-10 ≥ 4 (for LMICs) and FGS-10 ≥ 5 (upper-middle- and high-income countries) as proxies of micronutrient adequacy of intake of 11 micronutrients, with a validation method based on single 24-h recalls and using a mean adequacy ratio of 0.60 as the cutoff [16]. Our study addressed the gap identified in this previous study, namely, to study the predictive performance of MDD-W-related cutoffs using datasets of usual intakes across LMICs and in various low-income settings where adolescents have lower micronutrient adequacy levels. Our study additionally examined younger children aged 5–10 y and conducted subgroup analysis by age group.

Our results show, however, that the performances of FGS-10 ≥ 5 in children and adolescents were weaker than that in nonpregnant women of reproductive age as a proxy indicator for an acceptable micronutrient intake adequacy. Indeed, FGS-10 ≥ 5 showed fair performances to predict MPA-11 ≥ 0.60 in 3 of 7 sites for children and adolescents. Although these results were similar to those obtained for pregnant women (good performances in 2 of 6 sites) [13], the same cutoff had fair or good performances in predicting MPA-11 ≥ 0.60 in 6 of 9 sites in the study on nonpregnant women (although PCC was not presented) [11]. Nevertheless, the lower predictive performances were mainly related to lower sensitivity (3 sites) that would translate into misclassifying children with adequate MPA as having inadequate MPA.

The comparison of the performance of FGS-10 ≥ 5 with other healthy diet metrics, such as the GDQS or the EAT-Lancet diet score among children and adolescents, in predicting micronutrient adequacy, has yet to be determined. In the case of comparable performances in predicting micronutrient adequacy, FGS-10 ≥ 5 presents the advantages of being quick to compile and very operational in large-scale surveys and low-resources contexts. However, it does not allow for gathering information on diet components associated with a higher risk of noncommunicable disease, nor does it account for the environmental sustainability of diets. In practice, trade-offs exist among the simplicity of compilation, the spectrum of applications, and the principal concern (e.g. undernutrition, overnutrition, noncommunicable diseases risk, and environmental sustainability). As a result, the most appropriate diet quality metric among validated metrics may vary by context and purpose (research compared with programmatic compared with policy).

This study has several strengths including geographical diversity (pooling data from urban and rural settings across LMICs in 3 continents), broad age range (including children and adolescents aged 4–15 y), and large sample sizes. In addition, unlike prior validation studies whose distribution of MPA restricted the analysis to the prediction of acceptable levels of micronutrient intake adequacy (≥0.60) [11,13], our study showed sufficient variability in micronutrient adequacy levels in most of the datasets, which allowed us to validate FGS-10 ≥ 5 as a proxy for good micronutrient adequacy of children and adolescents.

Some limitations require consideration. Regarding external validity, despite the geographically diverse settings, the predictive performance was not assessed for the breadth of dietary patterns in LMICs. None of the included datasets were nationally representative. Additionally, some datasets were older than 10 y (Uganda, Zambia, Maharashtra, and Ecuador for instance). Therefore, different, specific or more recent dietary patterns may influence our site-specific results. Another limitation was that FGS-10 was calculated using quantitative 24-h recall data or observed weighed records in all sites. Although in reality, for nutrition programs or interventions, the calculation of FGS is generally made using a qualitative food group-based instrument [17]. This raises the question as to whether MDD-W indicators obtained from qualitative recall instruments are good proxy indicators for micronutrient adequacy. Indeed, qualitative recalls have been shown to misreport some food groups compared with quantitative methods, whether using open recalls or list-based questionnaires [[46], [47], [48], [49]]. In addition, primary studies validating MDD-W indicators obtained from qualitative questionnaires against micronutrient intake adequacy have shown mixed discriminatory performances [47,48]. Therefore, along with carefully developing context-specific food lists, addressing respondent biases and conducting extensive enumerator training to avoid misclassification of foods may improve the accuracy of measurement of MDD-W using qualitative questionnaire [46]. Finally, the effect of the age of respondents on the capacity to accurately report the consumption of food groups remains to be assessed, as for all dietary intake recall methods [7,50].

In conclusion, the continuous FGS-10 and dichotomous FGS-10 ≥ 5 are simple population proxy indicators of adequacy of micronutrient intake in children and adolescents aged 4–15 y in LMICs. Both indicators can be used to assess performances of diet-related policies and programs in this population. However, more research is needed to understand how best to operationalize data collection of simple food group intake as accurately as possible in children and adolescents.

## Authors contributions

The authors’ responsibilities were as follows – AG, LD, EBecquey, LH: designed research; JEA, DKO, EBoy, CL, AMO-A: provided the datasets; LD: analyzed data and conducted statistical analysis; LD, EBecquey, AG, LH: drafted the figures, tables, and manuscript, and the other authors provided critical review; and all authors: commented on drafts and read and approved the final manuscript.

## Data availability

Data analyzed in this study and scripts will be made available upon request and approval by the authors.

## Funding

Funding for this work was provided by the Bill & Melinda Gates Foundation (grant no. OPP1149709) and the World Food Programme (Service contract no. WFP/ITHQ/2021/4800337360).

## Conflict of interest

The authors report no conflicts of interest.
