# Supplementary material for: The Minimum Dietary Diversity For Women Indicator Can Be Extended To Children And Adolescents aged 4-15 Years As A Proxy Population Indicator For Good Micronutrient Adequacy Of Diets In Low- and Middle-Income Countries
Source: Curr Dev Nutr. 2024 Nov 20;9(1):104508. doi: 10.1016/j.cdnut.2024.104508 (PMC11719312; doi:10.1016/j.cdnut.2024.104508)
Supplement: multimedia component 1 [file mmc1.docx]

**Online supplementary material**

**Supplemental table 1:** Frequency of consumption of food groups and mean gram intakes among consumers (at least 15 grams).^1^

|  |  | **Starchy staples** | **Beans and peas** | **Nuts and seeds** | **Dairy** | **Flesh foods** | **Eggs** | **Dark green leafy vegetables** | **Vitamin A-rich vegetables and fruits** | **Other vegetables** | **Other fruits** |
| --- | --- | --- | --- | --- | --- | --- | --- | --- | --- | --- | --- |
| **Accra1, n=755** | % | 96 | 21 | 15 | 6.4 | 83 | 41 | 17 | 18 | 79 | 8.5 |
|  | Mean ± SD | 450 ± 319 | 67 ± 77 | 55 ± 38 | 65 ± 75 | 157 ± 127 | 65 ± 49 | 36 ± 49 | 109 ± 140 | 61 ± 79 | 295 ± 324 |
|  |  |  |  |  |  |  |  |  |  |  |  |
| **Accra2, n=71** | % | 100 | 55 | 14 | 20 | 85 | 37 | 14 | 11 | 73 | 18 |
|  | Mean ± SD | 626 ± 302 | 168 ± 167 | 106 ± 204 | 74 ± 57 | 131 ± 96 | 65 ± 34 | 55 ± 59 | 165 ± 144 | 110 ± 75 | 264 ± 225 |
|  |  |  |  |  |  |  |  |  |  |  |  |
| **BF1, n=237** | % | 100 | 30 | 40 | 11 | 55 | 2.0 | 27 | 12 | 92 | 18 |
|  | Mean ± SD | 455 ± 342 | 66 ± 58 | 54 ± 39 | 73 ± 91 | 62 ± 51 | 27 ± 8.4 | 78 ± 73 | 102 ± 93 | 136 ± 120 | 122 ± 110 |
|  |  |  |  |  |  |  |  |  |  |  |  |
| **BF2, n=2586** | % | 100 | 27 | 32 | 4.0 | 17 | 1.0 | 52 | 8.0 | 50 | 8.0 |
|  | Mean ± SD | 1069 ± 469 | 160 ± 155 | 159 ± 193 | 242 ± 167 | 97 ± 127 | 69 ± 49 | 138 ± 169 | 218 ± 187 | 121 ± 135 | 152 ± 141 |
|  |  |  |  |  |  |  |  |  |  |  |  |
| **Ecuador, n=3550** | % | 99 | 34 | 1.0 | 78 | 91 | 23 | 7.0 | 23 | 60 | 62 |
|  | Mean ± SD | 477 ± 240 | 62 ± 53 | 35 ± 27 | 278 ± 160 | 123 ± 84 | 59 ± 40 | 33.4 ± 20 | 96 ± 89 | 91 ± 75 | 152 ± 144 |
|  |  |  |  |  |  |  |  |  |  |  |  |
| **Maharashtra, n=219** | % | 99 | 52 | 49 | 86 | 1.8 | 5.5 | 19 | 0.46 | 47 | 11 |
|  | Mean ± SD | 121 ± 63 | 37 ± 28 | 33 ± 22 | 240 ± 207 | 59 ± 35 | 62 ± 38 | 29 ± 24 | 29 ± 0 | 44 ± 32 | 63 ± 40 |
|  |  |  |  |  |  |  |  |  |  |  |  |
| **Malawi, n=3605** | % | 99 | 39 | 10 | 1.0 | 36 | 5.0 | 34 | 59 | 59 | 9.0 |
|  | Mean ± SD | 292 ± 211 | 123 ± 149 | 56 ± 88 | 127 ± 124 | 72 ± 155 | 77 ± 43 | 71 ± 97 | 106 ± 114 | 97 ± 155 | 211 ± 218 |
|  |  |  |  |  |  |  |  |  |  |  |  |
| **Uganda, n=367** | % | 100 | 37 | 52 | 15 | 41 | 9.0 | 29 | 41 | 68 | 53 |
|  | Mean ± SD | 512 ± 265 | 67 ± 53 | 75 ± 71 | 153 ± 141 | 99 ± 84 | 48.5 ± 32 | 44 ± 30 | 175 ± 200 | 70 ± 64 | 141 ± 119 |
|  |  |  |  |  |  |  |  |  |  |  |  |
| **Zambia, n=134** | % | 100 | 40 | 56 | 4.0 | 40 | 10 | 63 | 28 | 92 | 23 |
|  | Mean ± SD | 360 ± 222 | 57 ± 52 | 92 ± 76 | 115 ± 155 | 44 ± 41 | 34 ± 14 | 49 ± 28 | 215 ± 124 | 64 ± 39 | 147 ± 149 |
| **Pooled, n=11524** | % | 99 | 34 | 16 | 28 | 52 | 12 | 28 | 30 | 60 | 27 |
|  | Mean ± SD | 545 ± 426 | 103 ± 125 | 106 ± 151 | 265 ± 166 | 111 ± 114 | 62 ± 43 | 95 ± 134 | 114 ± 124 | 96 ± 120 | 160 ± 159 |

^1^Values are mean ± SD (standard deviation) of grams, percentages (%) or frequencies (n). The 15 grams limit was applied at the individual food level.

**Supplemental Figure 1:** Correlation between FGS-10 and MPA-11 not adjusting for energy intake (Panel A) and adjusting for energy intake (Panel B).^1^


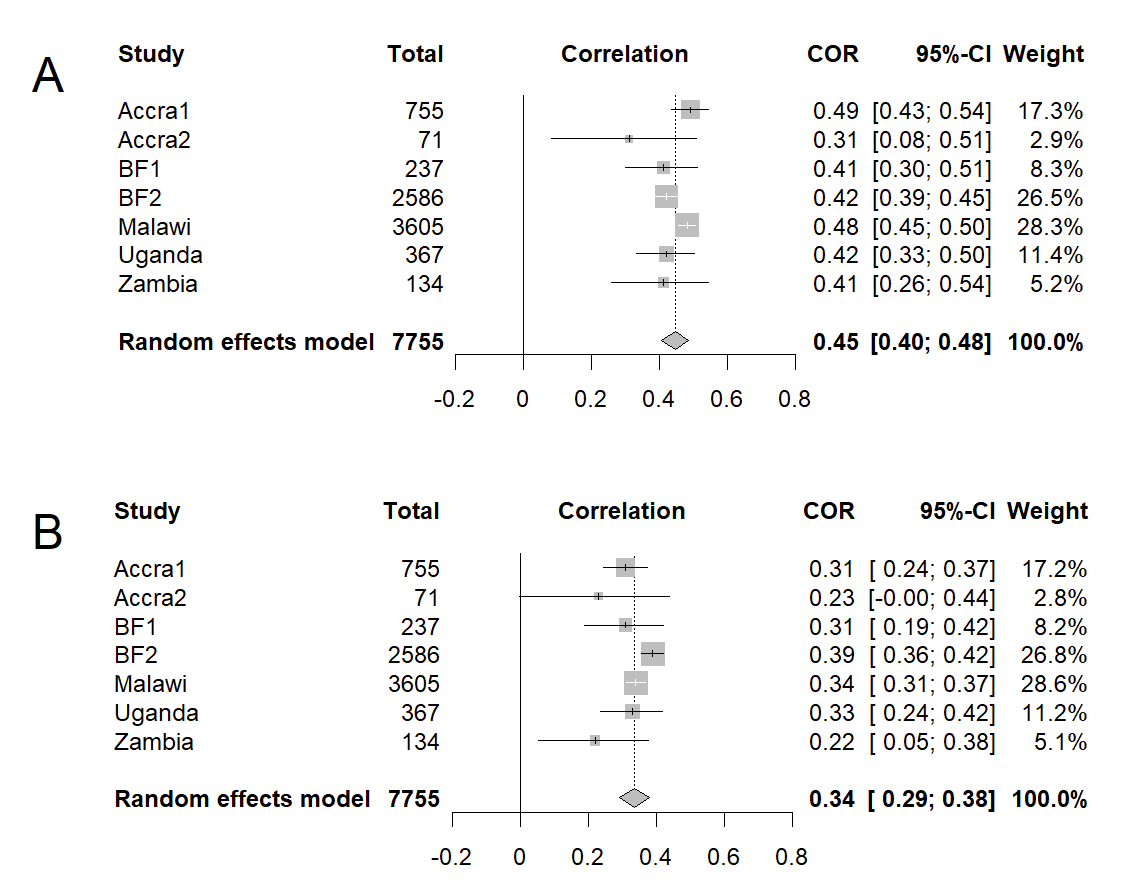


^1^Values are Spearman’s rank correlation coefficients, pooled correlation coefficients and confidence intervals of the pooled coefficients. Values unadjusted for energy intake are partial Spearman’s rank correlation coefficients. CI, Confidence interval.

**
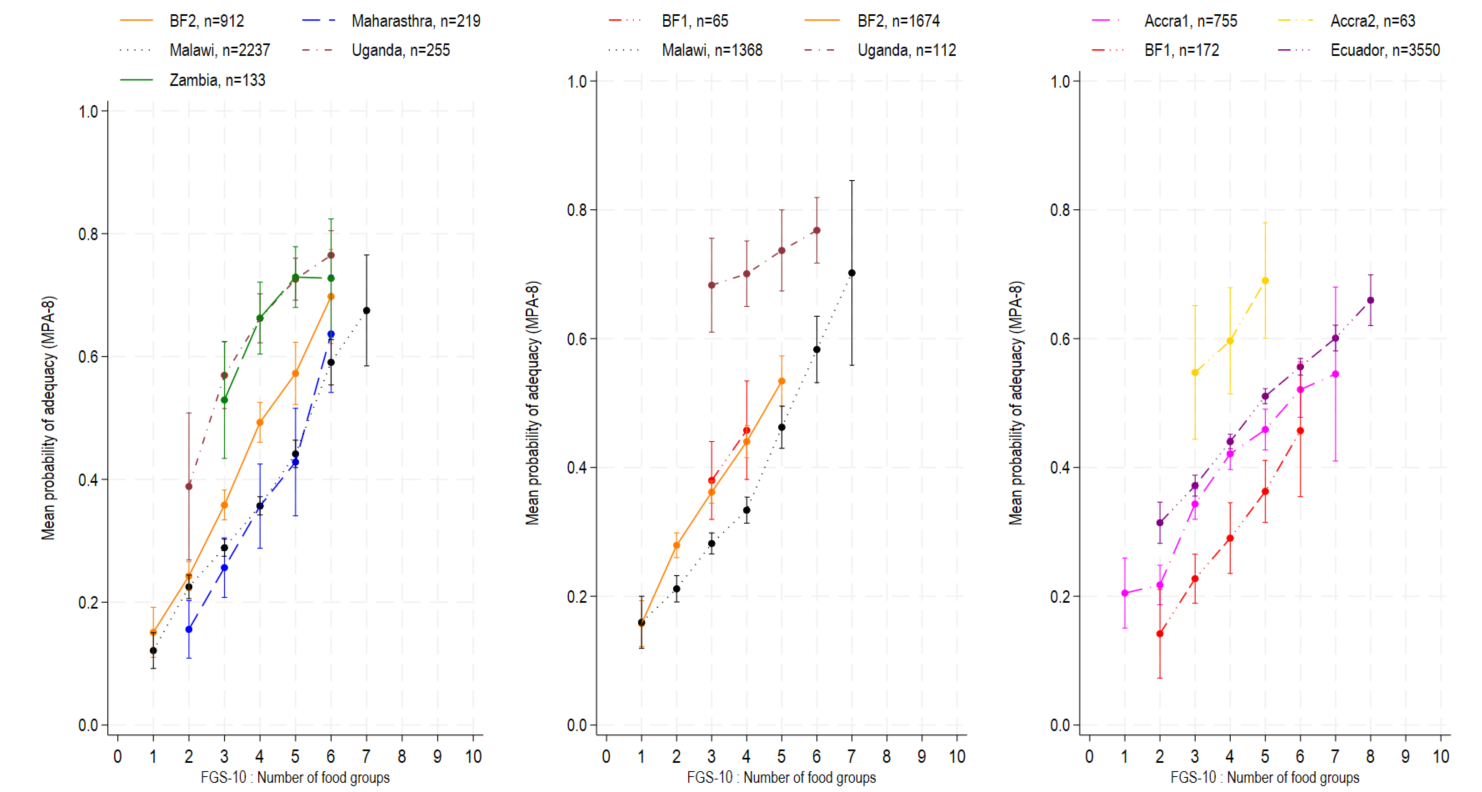
Supplemental Figure 2:** Mean probability of adequacy calculated on 8 micronutrients (MPA-8) by FGS-10 score, for children/ adolescents aged 4-4.9 years (panel A), 5-9.9 years (panel B) and 10-15 years (panel C). Values are mean ± SEM (Standard Error of the Mean). Food group scores with less than 10 observations were excluded. Accra1 = 755, Accra2 = 71, BF1 = 237, BF2 = 2586, Ecuador = 3550, Maharashtra = 219, Malawi = 3605, Uganda = 367, Zambia = 134.

**C**

**B**

**A**

**Supplemental Figure 3:** Correlation between FGS-10 and MPA-8 adjusting for energy intake among children/adolescents aged 4-4.9 years (Panel A), 5-9.9 years (panel B) and 10-15 years (panel C)^1^.

**A**


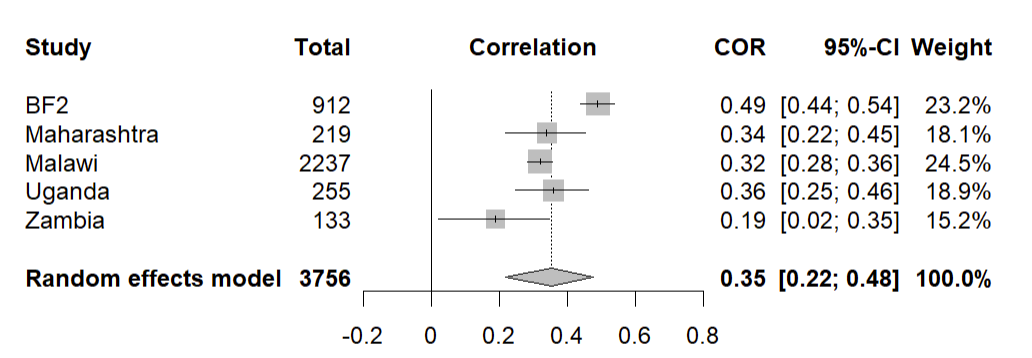


**B**


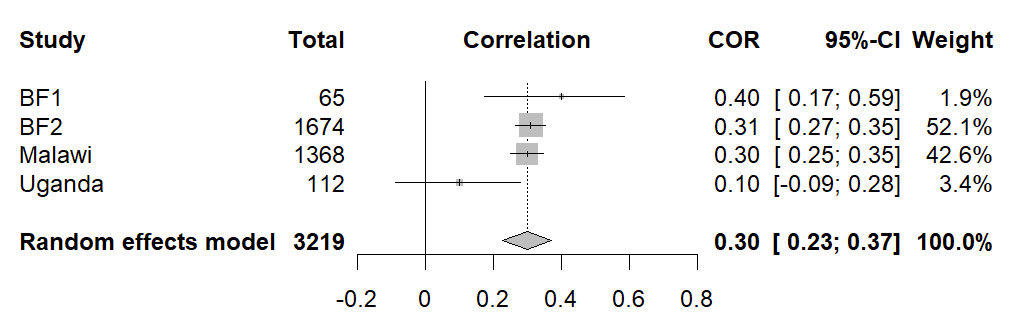


**C**


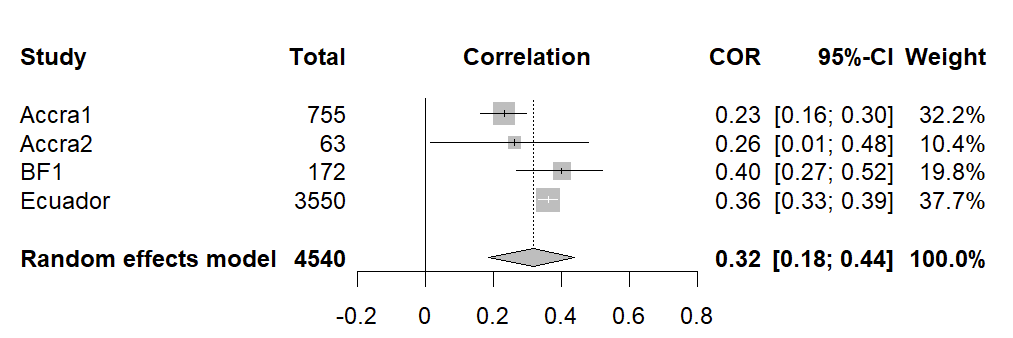


^1^Accra2 removed from analyses in panel B as there were only 8 observations.

**Supplemental Figure 4:** Receiver operating characteristic curves of FGS-10 predicting MPA-11 ≥ 0.60 (A) and MPA-11 ≥ 0.80 (B) by study site.


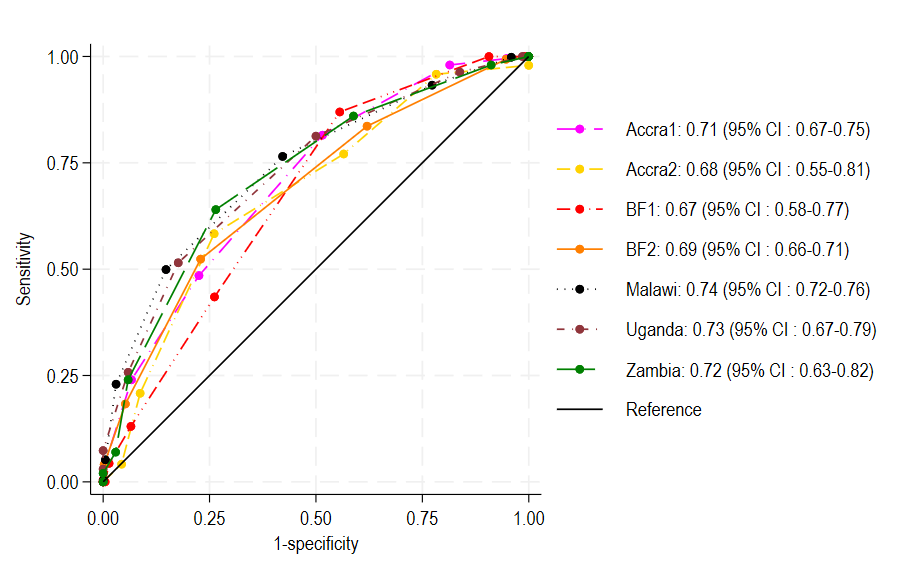


**A**


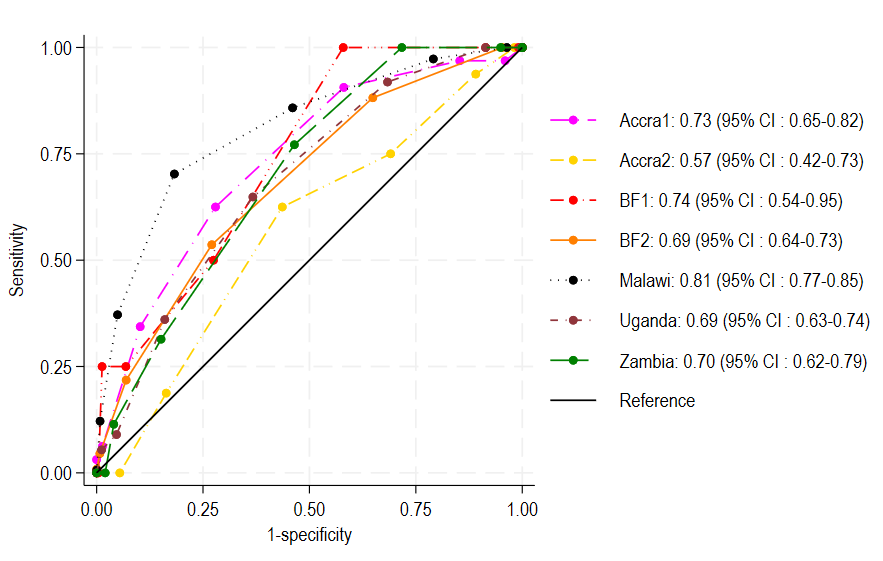


**B**

**Supplemental table 2:** Summary of FGS-10 cut-offs characteristics relative to predicting MPA-11≥0.60.^1^

|  |  | **Sensitivity, %** | **Specificity, %** | **Youden index** | **PCC (%) / Pooled AUC** |
| --- | --- | --- | --- | --- | --- |
| **Accra1** |  |  |  |  |  |
|  | FGS-10≥4 | 82 | 49 | 0.30 | 57 |
|  | FGS-10≥5 (MDD-W) | 49 | 78 | 0.26 | 70 |
|  | FGS-10≥6 | 24 | 93 | 0.17 | 75 |
| **Accra2** |  |  |  |  |  |
|  | FGS-10≥4 | 77 | 44 | 0.21 | 66 |
|  | FGS-10≥5 (MDD-W) ^2,4^ | 58 | 74 | 0.32 | 63 |
|  | FGS-10≥6 | 21 | 91 | 0.12 | 44 |
| **BF1** |  |  |  |  |  |
|  | FGS-10≥4 | 87 | 44 | 0.31 | 49 |
|  | FGS-10≥5 (MDD-W) | 44 | 74 | 0.17 | 71 |
|  | FGS-10≥6 | 13 | 94 | 0.07 | 86 |
| **BF2** |  |  |  |  |  |
|  | FGS-10≥4 ^2,4^ | 52 | 77 | 0.30 | 73 |
|  | FGS-10≥5 (MDD-W) | 18 | 95 | 0.13 | 81 |
|  | FGS-10≥6 | 4.1 | 100 | 0.04 | 83 |
| **Malawi** |  |  |  |  |  |
|  | FGS-10≥4 ^2^ | 77 | 58 | 0.34 | 61 |
|  | FGS-10≥5 (MDD-W) ^2,4^ | 50 | 85 | 0.35 | 80 |
|  | FGS-10≥6 | 23 | 97 | 0.20 | 85 |
| **Uganda** |  |  |  |  |  |
|  | FGS-10≥4 ^2,4^ | 81 | 50 | 0.31 | 76 |
|  | FGS-10≥5 (MDD-W) | 52 | 82 | 0.34 | 57 |
|  | FGS-10≥6 | 26 | 94 | 0.20 | 38 |
| **Zambia** |  |  |  |  |  |
|  | FGS-10≥4 | 86 | 41 | 0.27 | 75 |
|  | FGS-10≥5 (MDD-W) ^2,4^ | 64 | 74 | 0.38 | 66 |
|  | FGS-10≥6 | 24 | 94 | 0.18 | 42 |
| **Pooled** |  |  |  |  |  |
|  | FGS-10≥4 ^2,4^ | 78 CI: 70-85 | 54 CI: 44-64 | 0.32 CI: 0.29-0.36 | 0.72 |
|  | FGS-10≥5 (MDD-W) | 48 CI: 36-61 | 82 CI: 73-89 | 0.30 CI: 0.23-0.37 | 0.72 |
|  | FGS-10≥6 | 20 CI: 12-30 | 96 CI: 92-99 | 0.16 CI: 0.10-0.22 | 0.61 |

^1^Values of sensitivity, specificity and correctly classified are percentages. BF, Burkina Faso; CI, Confidence Interval; FGS-10, Food group score based on the minimum dietary diversity for women (MDD-W) indicator guidelines; MPA-11, Mean Probability of adequacy calculated over 11 micronutrients; PCC, percentage of correct classification; Pooled AUC, Area under the curve obtained by plotting sensitivities and false positive rates of all sites.

^2^ Cut-off with fair predictive performances, defined as PCC>60% and both specificity and sensitivity > 50%, with at least one of them being >60%.

^3^ Cut-off with predictive good performances, defined as PCC>70%, and both sensitivity and specificity >60%.

^4^ Optimal cut-off, defined as cut-off with highest Youden index (Sensitivity + Specificity – 1).

**Supplemental table 3:** Summary of FGS-10 cut-offs characteristics relative to predicting MPA-11≥0.80^1^.

|  |  | **Sensitivity, %** | **Specificity, %** | **Youden index** | **PCC (%) / Pooled AUC** |
| --- | --- | --- | --- | --- | --- |
| **Accra1** |  |  |  |  |  |
|  | FGS-10≥4 | 91 | 42 | 0.33 | 44 |
|  | FGS-10≥5 (MDD-W) ^3,4^ | 63 | 72 | 0.35 | 72 |
|  | FGS-10≥6 | 34 | 90 | 0.24 | 87 |
| **Accra2** |  |  |  |  |  |
|  | FGS-10≥4 | 75 | 31 | 0.06 | 41 |
|  | FGS-10≥5 (MDD-W) | 63 | 56 | 0.19 | 58 |
|  | FGS-10≥6 | 19 | 84 | 0.02 | 69 |
| **BF1** |  |  |  |  |  |
|  | FGS-10≥4 ^2,4^ | 50 | 73 | 0.23 | 72 |
|  | FGS-10≥5 (MDD-W) | 25 | 93 | 0.18 | 92 |
|  | FGS-10≥6 | 25 | 99 | 0.24 | 98 |
| **BF2** |  |  |  |  |  |
|  | FGS-10≥4 ^2,4^ | 54 | 73 | 0.27 | 72 |
|  | FGS-10≥5 (MDD-W) | 22 | 93 | 0.15 | 90 |
|  | FGS-10≥6 | 4.5 | 99 | 0.04 | 95 |
| **Malawi** |  |  |  |  |  |
|  | FGS-10≥4 | 86 | 54 | 0.40 | 55 |
|  | FGS-10≥5 (MDD-W) ^3,4^ | 70 | 82 | 0.52 | 81 |
|  | FGS-10≥6 | 37 | 95 | 0.32 | 93 |
| **Uganda** |  |  |  |  |  |
|  | FGS-10≥4 | 92 | 32 | 0.24 | 50 |
|  | FGS-10≥5 (MDD-W) ^2,4^ | 65 | 63 | 0.28 | 64 |
|  | FGS-10≥6 | 36 | 84 | 0.20 | 70 |
| **Zambia** |  |  |  |  |  |
|  | FGS-10≥4 | 100 | 28 | 0.28 | 47 |
|  | FGS-10≥5 (MDD-W) | 77 | 54 | 0.31 | 60 |
|  | FGS-10≥6 | 31 | 85 | 0.16 | 71 |
| **Pooled** |  |  |  |  |  |
|  | FGS-10≥4 | 85 CI: 75-93 | 44 CI: 33-57 | 0.30 CI: 0.23-0.37 | 0.70 |
|  | FGS-10≥5 (MDD-W) ^2,4^ | 60 CI: 44-75 | 73 CI: 60-84 | 0.34 CI: 0.23-0.43 | 0.72 |
|  | FGS-10≥6 | 27 CI: 15-41 | 92 CI: 85-97 | 0.19 CI: 0.10-0.30 | 0.65 |

^1^Values of sensitivity, specificity and correctly classified are percentages. BF, Burkina Faso; CI, Confidence Interval; FGS-10, Food group score based on the minimum dietary diversity for women (MDD-W) indicator guidelines; MPA-11, Mean Probability of adequacy calculated over 11 micronutrients; PCC, Percentage of correct classification; Pooled AUC, Area under the curve obtained by plotting sensitivities and false positive rates of all sites.

^2^ Cut-off with fair predictive performances, defined as PCC>60% and both specificity and sensitivity > 50%, with at least one of them being >60%.

^3^ Cut-off with predictive good performances, defined as PCC>70%, and both sensitivity and specificity >60%.

^4^ Optimal cut-off, defined as cut-off with highest Youden index (Sensitivity + Specificity – 1).
